# Environmental and Health Care Personnel Sampling and Unobserved *Clostridium difficile* Transmission in ICU

**DOI:** 10.1001/jamanetworkopen.2025.2787

**Published:** 2025-04-04

**Authors:** Lindsay T. Keegan, Windy Tanner, Brian Orleans, Rachel B. Slayton, John A. Jernigan, L. Clifford McDonald, Judith Noble-Wang, Molly Leecaster, Candace Haroldsen, Karim Khader, Damon J.A. Toth, Tierney O’Sullivan, Matthew H. Samore, William Brazelton, Michael Rubin

**Affiliations:** 1 Division of Epidemiology, Department of Internal Medicine, University of Utah, Salt Lake City; 2 Salt Lake City Veterans Healthcare Administration IDEAS Center, Salt Lake City, Utah; 3 Yale University, Department of Epidemiology of Microbial Diseases, Yale School of Public Health, New Haven, Connecticut; 4 Huntsman Cancer Institute, University of Utah, Salt Lake City; 5 US Centers for Disease Control and Prevention Division of Healthcare Quality Promotion, Atlanta, Georgia; 6 Department of Population Health Sciences, University of Utah, Salt Lake City; 7 School of Biological Sciences, University of Utah, Salt Lake City

## Abstract

**Importance:**

*Clostridioides difficile* is among the most prevalent health care–associated pathogens worldwide. Controlling it remains a critical challenge, due in part to spore viability on surfaces.

**Objective:**

To quantify transmission of *C difficile* within health care facilities and evaluate the roles of environmental surfaces and health care personnel (HCP) hands in *C difficile* movement.

**Design, Setting, and Participants:**

In 2018, a 13-week longitudinal, observational study was conducted in 2 intensive care units (ICUs) in Utah with daily culture-based sampling of patient body sites, room environmental surfaces, HCP hands, and shared environmental surfaces. Both toxigenic and nontoxigenic *C difficile* strains were selected for whole genome sequencing and included in the analysis. Data were analyzed from September 2021 to September 2024.

**Main Outcomes and Measures:**

The primary outcome was the identification of transmission clusters based on genomic relatedness between isolates from patients, environmental surfaces, and HCP hands. Clusters were defined as isolates with 2 or fewer single nucleotide variants between them.

**Results:**

Of the 278 unique ICU admissions, 177 patients consented to body site sampling and were sampled. Along with these, environment surfaces and HCP hands were sampled daily for all occupied rooms, leading to 7000 total samples. Sampling patients, their environment, and HCP hands revealed that nearly 8% of all patients had *C difficile* linked to other admissions and 57% of transmission clusters bridged nonoverlapping patient-stays. Including environmental surfaces and HCP hands, a 3.6-fold higher *C difficile* movement was identified than with patient sampling alone, highlighting environmental surfaces as reservoirs.

**Conclusions and Relevance:**

These results challenge the idea that nosocomial transmission is not a primary source of acquisition and underscore the importance of hand hygiene and environmental decontamination. This study reinforces the need to include environmental surfaces and HCP hands in future work characterizing the burden of nosocomial transmission. Understanding the transmission pathways of *C difficile* within health care facilities, particularly the roles of environmental surfaces and HCP hands, is critical to improving infection control measures.

## Key Points

**Question:**

What is the role of environmental surfaces and health care personnel (HCP) hands in the transmission of *Clostridioides difficile* within health care facilities?

**Findings:**

This cohort study of 278 unique intensive care units (ICU) admissions, 177 patients, and 7000 samples in 2 ICUs revealed that nearly 8% of patients had *C difficile* linked to other admissions. Including environmental surfaces and HCP hands, 3.6-fold higher *C difficile* movement was identified than with patient sampling alone.

**Meaning:**

These findings suggest that environmental surfaces and HCP hands play critical roles in *C difficile* transmission. These findings challenge the idea that nosocomial transmission is not a primary source of acquisition and underscore the importance of hand hygiene and environmental decontamination.

## Teaser

This cohort study evaluates transmission of *Clostridium difficile* within intensive care units (ICUs) and assesses the roles of environmental surfaces and health care personnel hands in *C difficile* movement.

## Introduction

*Clostridioides difficile* infections (CDI) are a leading cause of health care–associated infections (HAI) worldwide, accounting for over 223 000 hospitalized cases and 12 800 deaths annually in the US.^1,2,3,4,5^ Identifying and mitigating transmission within health care facilities (HCF) remains a critical challenge to controlling CDI. Previous studies revealed that most new CDls cannot be linked to other symptomatic CDIs^3,6^; similarly, recent studies that include asymptomatic toxigenic *C difficile* found that carriers also infrequently cause new symptomatic CDIs.^7,8,9,10^ Since most genomic epidemiologic studies include only patient sampling, one potential understudied source for unlinked acquisitions is *C difficile* spores that persist on environmental surfaces and evade standard infection control measures.^2,11^ Understanding the potential of these surfaces to act as reservoirs in HCFs is crucial for developing effective infection control strategies.^3,6,7,8,11,12^

A significant challenge in identifying environmental reservoirs in HAIs is the overwhelming number of potential fomites.^13,14,15^ In the intensive care unit (ICU), where direct patient-to-patient contact is infrequent, it follows that the transmission of HAIs predominantly occurs through intermediary vectors. Although health care personnel (HCP) hands are frequently implicated as vectors, evidence supporting the extent to which they mediate pathogen movement remains inconclusive.^16^

In this study, we quantified rates of *C difficile* spread across 2 ICUs and add critical data to contextualize the role environmental surfaces and HCP hands play in patient-to-patient transmission.^17,18^ To describe transmission dynamics, we differentiate between transmission pathways (the route *C difficile* takes between patients^19^), and pathogen movement (the spreading of *C difficile* between different sites and surfaces). We analyzed the temporal and spatial contributions of patients, the environment, and HCP hands to transmission in 2 different ICUs. While previous studies have largely focused on toxigenic *C difficile*, we consider both toxigenic and nontoxigenic strains, providing a broader perspective on transmission dynamics within HCFs, particularly transmission that may occur below the detection limit of most surveillance systems. This comprehensive approach allowed us to identify previously undetected patterns of *C difficile* spread, highlighting the importance of measuring diverse sources in infection control.

## Methods

All protocols were approved by the University of Utah institutional review board. Patients were provided with an informational brochure and provided verbal assent. Demographic data were not collected to protect patients’ identities. The study followed Strengthening the Reporting of Observational Studies in Epidemiology (STROBE) reporting guidelines.

### Sample Collection

We conducted a daily, longitudinal study of HAIs in 2018, collecting microbiologic samples in 2 ICUs in Utah: a 20-bed cardiovascular ICU in a 550-bed tertiary acute care hospital (Hospital A) and a 10-bed medical and surgical ICU in an 80-bed acute care hospital (Hospital B). Hospital A was sampled daily for 8 weeks from study day 1 through 55, and Hospital B was sampled daily for 5 weeks from study day 93 through 127. These ICUs are in the same city and occasionally share HCPs.

If consented, patients were sampled from up to 3 patient body sites: the axilla, the groin, and either perianal region or stool. Room environments were sampled from 3 surfaces: patient touch surfaces, HCP touch surfaces, and toilet surfaces (details in eMethods in Supplement 1).^20^ Hands or gloves (if worn) of HCPs who cared for the patient were sampled upon room exit and before hand hygiene or glove removal. At least 1 HCP hand sample was collected from each occupied room daily. Shared surfaces were sampled daily.

We assigned each new patient a unique occupant stay identification, associated with samples collected during their stay. This followed the patient throughout their stay, including room changes and for 24 hours following transfer to another ward (details in eMethods in Supplement 1).

### Multilocus Sequence Typing and Whole-Genome Sequencing

Morphologically distinct *C difficile* colonies were isolated from cycloserine cefoxitin fructose agar with horse blood and taurocholate agar for species identification by Matrix-assisted laser desorption ionization–time of flight (MALDI-TOF) (Bruker Biotyper CA System) (see eMethods in Supplement 1). All *C difficile* isolates identified by MALDI-TOF were subjected to both to multilocus sequence typing (MLST) and whole-genome sequencing (WGS).^21^ pubMLST was used to determine sequence type.^22^ Isolates were selected irrespective of toxin production.

WGS was completed by the Huntsman Cancer Institute/UHealth High-Throughput Genomics Laboratory using a Nova-Seq (Illumina) platform.^21^ DNA was extracted from patient multidrug-resistant organism isolates using the DNEasy Ultraclean Microbial kit (Qiagen). Nextera (Illumina) DNA Flex libraries with a 350 to 400 base pair (bp) insert size were prepared and included 8 to 12 polymerase chain reaction cycles of tagmented DNA. Libraries were subjected to 150-bp paired-end sequencing using the Illumina NovaSeq6000 located in the Huntsman Cancer Institute/UHealth High-Throughput Genomics Laboratory. The NovaSeq XP workflow was used to multiplex 384 samples for each of the 2 lanes of an S prime Flow cell (768 isolates total), generating 600 to 800M read-pairs, giving an estimated 50 ×  to 60 × coverage of each genome (see eMethods in Supplement 1 for bioinformatic details).^23,24,25,26,27^ Toxin genes were detected with Basic Local Alignment Search Tool Protein using the complete *tcdA* and *tcdB* genes against predicted protein sequences from the genome assemblies.^28^

### Acquisition Analysis

We applied rule-based criteria without consideration of genomics and included patients if they were sampled within 2 days of admission and again on day 3 or later. Importation was defined as a positive body site sample within 2 days of admission, and acquisition was defined as a combination of negative admission sample(s) and positive follow-up sample(s).

### Genomic Analyses

A whole-genome alignment containing core single nucleotide variants (SNV) as well as invariant sites was generated by the R package Snippy, version 4.6.0, for each clade.^29^ SNVs were identified by aligning the quality-trimmed sequence reads for each *C difficile* genome to a reference genome with Snippy. A single, high-quality reference genome was selected from the National Center for Biotechnology Information database for each *C difficile* clade (clade 1 = NZ_CP019870.1, clade 2 = NC_013316.1, and clade 4 = FN668375.1). No isolates from clade 3, 5, or the cryptoclades were obtained during this study.^30,31,32,33,34,35,36,37^

### Statistical Analysis

To establish a threshold indicative of recent transmission, we analyzed the pairwise genomic distances between all patient body site isolates collected from the same occupant stay, using snp-dists.^3,6,7,26,27,36^ We compared all isolates in the study and defined transmission to be any pair or group of isolates from different occupant stays that were separated by no more than the threshold and used ggraph in R version 4.2.0 (R Project for Statistical Computing) to visualize these results.^37,38^ We conducted a sensitivity analysis around the clustering threshold (eResults in Supplement 1). Data were analyzed from September 2021 to September 2024.

## Results

### Cohort Description

We collected 7000 samples across 278 unique admissions, with 177 patients consenting to patient body site sampling (eFigure 1 in Supplement 1). We recovered 178 *C difficile* isolates from 161 samples belonging to 35 unique occupant stays, representing 25 of 278 admissions (12.6%). There were 46 isolates from patient body sites, 87 isolates from patient rooms, 1 isolate from a shared environmental surface, and 44 isolates from HCP hands (eFigure 2 in Supplement 1).

We found the combined period prevalence of *C difficile* (toxigenic and nontoxigenic) was 6.78% (12 of 177 body sites) among patient body sites and 7.30% (20 of 274 surfaces) for environmental surfaces across both hospitals (eFigure 3 in Supplement 1). Similarly, the period prevalence of toxigenic *C difficile* alone was 1.69% (3 of 178 patients) among patient body sites and 3.28% (9 of 274 surfaces) for environmental surfaces across both hospitals. On average, we recovered *C difficile* from 1.89% (95% CI, 1.59%-2.20%) of HCP hands per occupied room per day. We found lower rates of toxigenic *C difficile* acquisition when compared with recent studies (0.56% vs 1%^7^), yet higher overall acquisition rates (1.68%) when including both toxigenic and nontoxigenic *C difficile*.

### Quantification of Sequence Type Diversity

**Figure 1.**
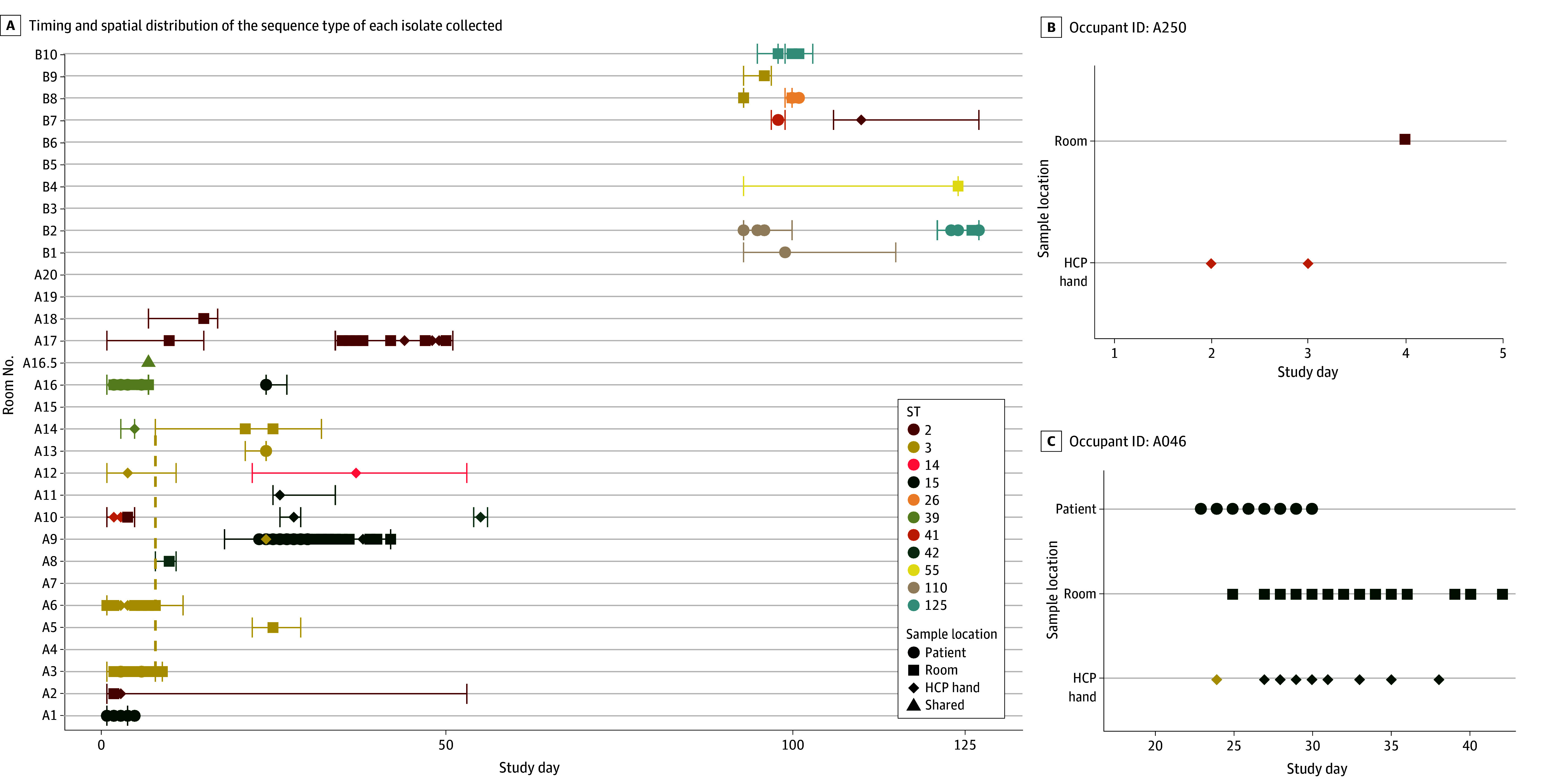
*C difficile* Sequence Type (ST) Diversity Within and Between Patients Over a 127-Day Period in 2018 A, Timing and spatial distribution of the sequence type of each isolate collected in the study alongside occupant stay admission and discharge time (vertical bars connected by a horizontal bar) with room transfers shown with a dotted vertical line. B, Occupant stay in room A10. C, Occupant stay in room A9. HCP indicates health care personnel.

We assessed the diversity of strains in our study at the sequence type level and found 11 distinct sequence types (Figure 1).^39^ Each hospital had isolates from 7 different sequence types; only 3 were found at both facilities (sequence type 2, 3, and 41). We observed 2 instances of isolates belonging to different sequence types from the same occupant stay (Figure 1B). In 1 instance, isolates from HCP hands belonged to 1 sequence type and an environmental isolate belonged to another; in the other, 2 sequence types were recovered from a single swab.

Thirty-five *C difficile* isolates (19.6%) had the *tcdA* and *tcdB* toxin loci (defined as toxigenic) and 143 isolates (79.9%) did not (defined as nontoxigenic). All toxigenic patient isolates were from hospital B, although hospital A had toxigenic environmental and HCP hand isolates. Only sequence type 3 included both toxigenic and nontoxigenic strains. We recovered isolates from clade 1 (156 of 179 isolates [87.1%]), clade 2 (4 isolates [2.2%]), and clade 4 (19 isolates [10.6%]) (eFigure 4 in Supplement 1). Toxigenic isolates were distributed across clades 1 and 2.

### Longitudinal Comparison of *C difficile* Diversity Across Scales

We compared the genetic diversity of isolates between occupant stays over time to elucidate the transmission dynamics across scales. We found 10 instances of pathogen spread around a patient’s room. Specifically, we recovered the same strain (defined as ≤2 SNVs between isolates) from at least 2 different sampling locations, including on different body sites, room surfaces, or a combination on the same or different day. This offers insight into either first or last steps in the transmission pathway: either patients shedding pathogen into their environment or patients acquiring it from their environment.

We primarily found that isolates from patient body sites were more closely related to isolates from their own room (median [IQR], 0 [0-0] SNVs) than to isolates from other patients or different rooms (median [IQR], 991 [973-1286] SNVs) (eFigure 5 in Supplement 1). We found differences in genetic similarity across sampling locations for patients on contact precautions compared with those not on contact precautions (eFigure 6 in Supplement 1), but isolates from patients on contact precautions were not genetically more similar to each other than isolates from patients on standard precautions.

### Assessment of *C difficile* Importation and Acquisition

**Figure 2.**
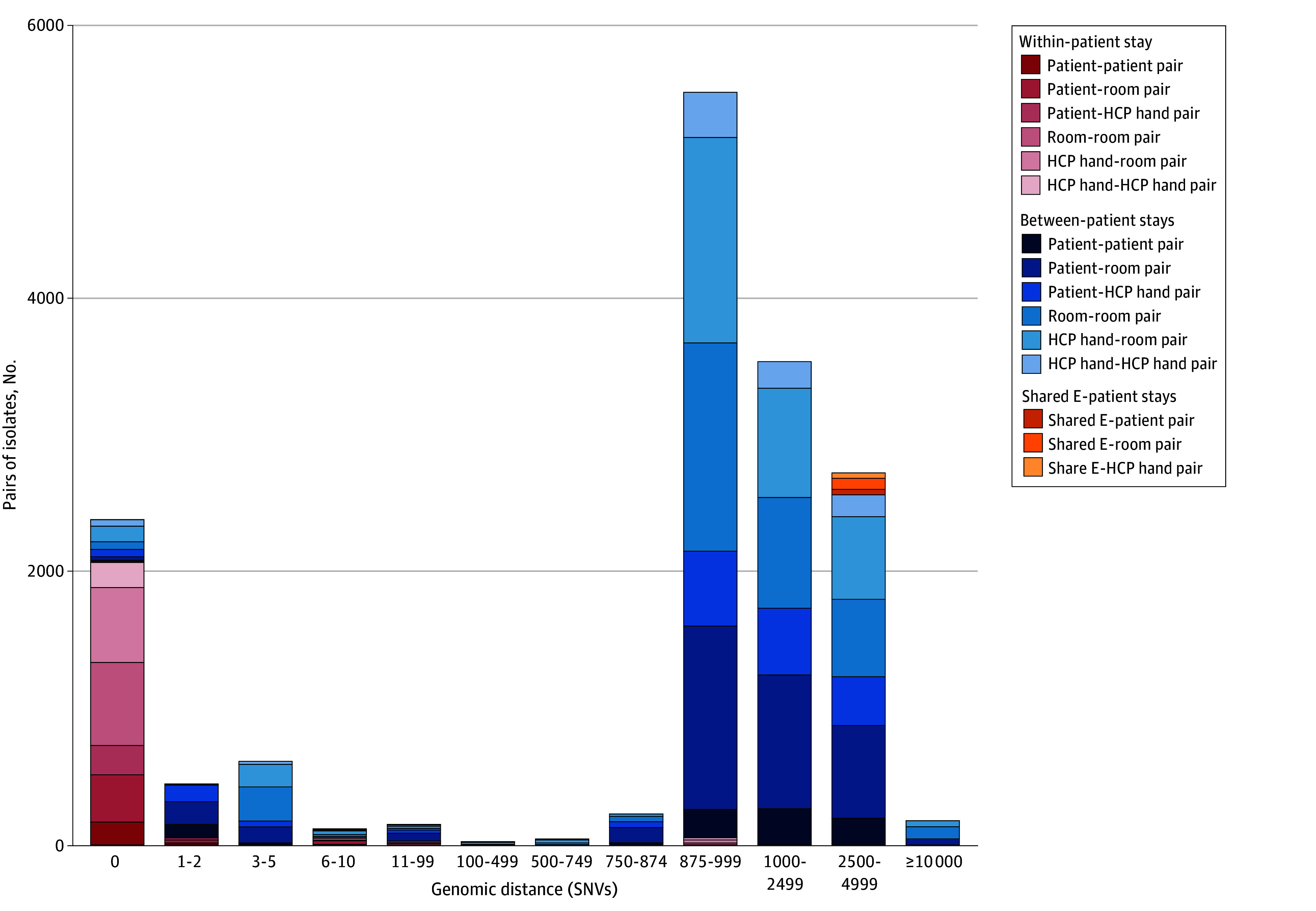
Comparison of Genomic Linkages Within and Between Hospitals and Occupant Stays Over a 127-Day Period in 2018 Plot of pairwise distance between all pairs (N=15,753) of isolates in the study, including both same patient and different patient as well as all combinations of sampling locations. For each isolate in the study, we compare the distance (in SNPs) between it and every other isolate in the study and color based on the occupant stay and sample location of the isolates. HCP indicates health care personnel; SNV, single nucleotide variant.

Nine patients met the criteria for assessing acquisition (8 patients were excluded for importing *C difficile:* 2 imported toxigenic and 5 nontoxigenic). Only 1 patient was found to have acquired toxigenic *C difficile*. Three other patients did not meet the criteria due to no sampling on admission, but nontoxigenic *C difficile* was recovered in later samples. Although body sites were not sampled on admission for these patients, *C difficile* was not recovered for multiple days of room environment or HCP hands samples before recovering the first patient isolate. We consider these potential acquisition events. The timing of samples and isolate recovery is shown in Figure 2. By including patient room surfaces and HCP hands, we were able to characterize the timing of contamination with respect to patient colonization. We found *C difficile* was recovered from room surfaces and HCP hand samples a mean (SD) of 0.8 (1.64) days and 1.5 (2.08) days, respectively, after the first *C difficile* isolate was recovered from the occupying patient (eFigure 7 in Supplement 1).

### Genomic Analysis of Pathogen Movement in the ICU

**Figure 3.**
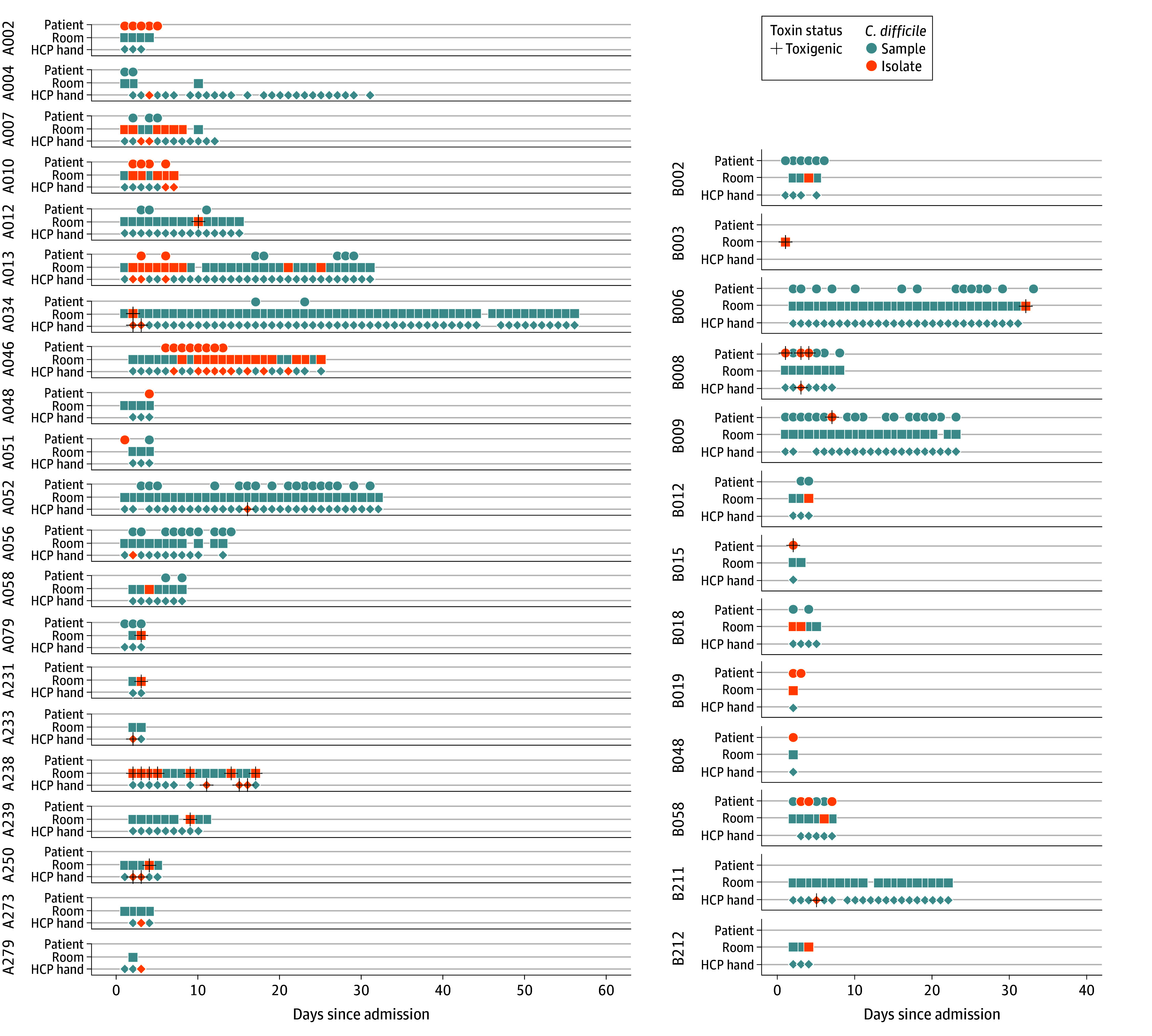
Daily Sampling by Room Location Over a 127-Day Period in 2018 Plot of the timing in days since admission for all of the isolates and samples from each patient with at least one isolate recovered by occupant stay identifier. Each point represents a sample collected. Each sub-plot represents an occupant stay (denoted on the left). HCP indicates health care personnel.

**Figure 4.**
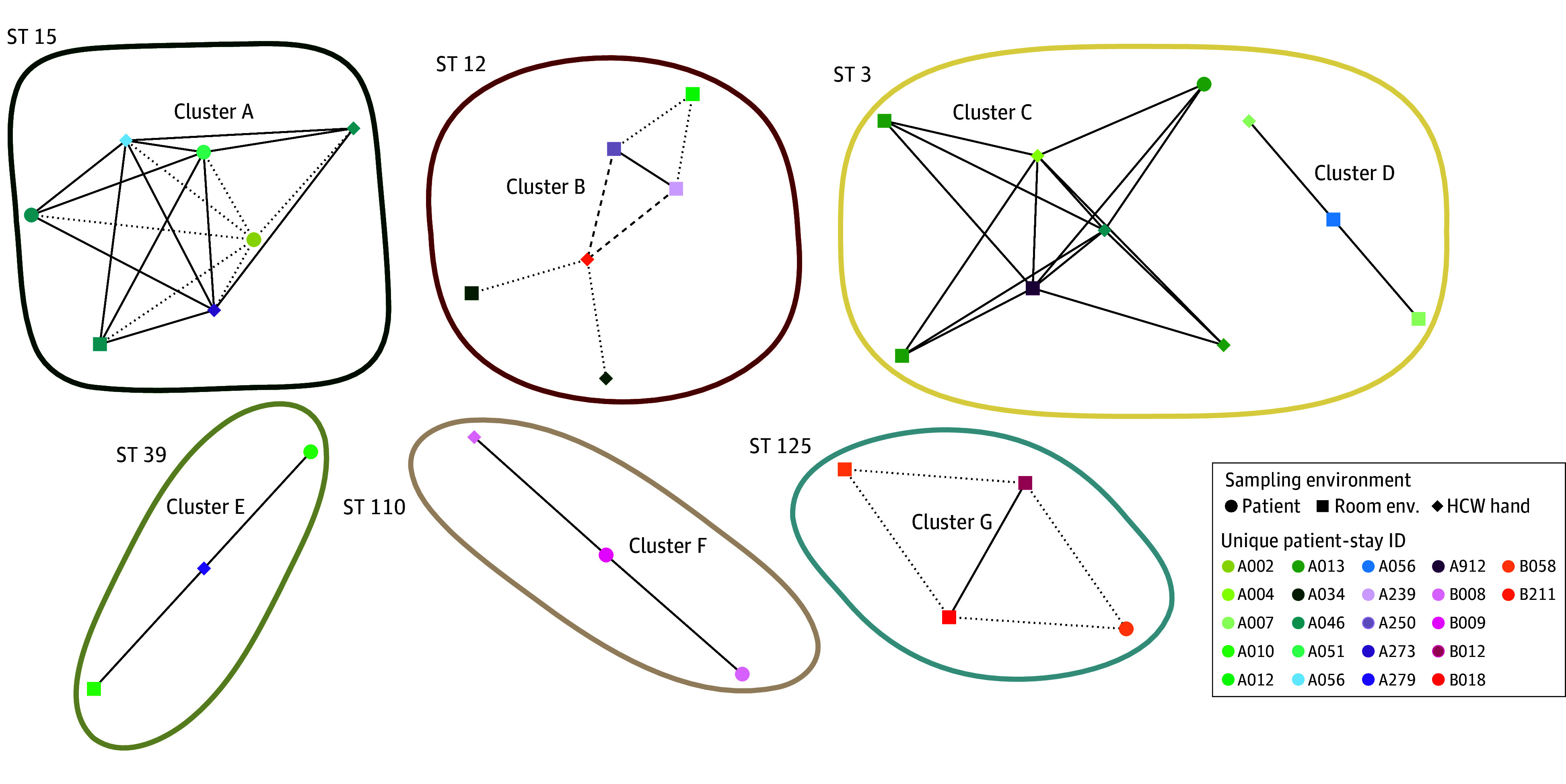
Transmission Networks Identified by Single Nucleotide Variant (SNV) Threshold Network plot of each transmission cluster where each node is colored by occupant stay and the shape represents all the isolates from that occupant stay from that sampling location. The number of genomic distance (in SNVs) between a pair of isolates is indicated by an edge, and the line type depicts the distance. Clusters are circled by sequence type and labeled within the circle. All isolates in clusters B and F are toxigenic. HCP indicates health care personnel; ID, identification; ST, sequence type.

We identified a natural transmission threshold of 2 or fewer SNVs, consistent with other studies (Figure 3).^6,7,25^ Applying our threshold, we identified 7 transmission clusters involving 22 unique occupant stays (Figure 4). In other words, 22 of 287 occupant stays (7.7%) were implicated in *C difficile* transmission. Two clusters (28.5%) involved toxigenic isolates, and all of the isolates in these 2 clusters were toxigenic. Among these transmission clusters, 2 (28.5%) included isolates from 2 distinct occupants’ body sites, suggesting patient-to-patient transmission (Figure 4); 1 of these patient-to-patient transmission clusters included only toxigenic isolates (cluster F) and 1 included only nontoxigenic isolates (cluster A). Two other clusters included an environmental or HCP hand isolate and a patient body site isolate from different occupant stays, representing either a patient acquiring from, or shedding into, the environment or HCP hands of another occupant stay. The remaining 3 clusters included isolates from environmental surfaces from multiple occupant stays, highlighting pathogen movement around a facility independent of patient involvement. Importantly, 5 of these transmission clusters (71.4%) would have been missed without the expanded sampling of environmental surfaces and HCP hands because they did not include patient body site isolates from multiple occupant stays.

Validating our acquisition analysis using WGS data revealed that the patient identified as having an acquisition—as well as two-thirds of the patients identified as potentially having an acquisition—clustered with other occupant stays, supporting our findings of within-ward transmission. The patient who acquired *C difficile* clustered with another patient, indicating likely patient-to-patient transmission, while the other 2 with potential acquisitions clustered with an environmental surface and HCP hand, respectively.

Most patient isolates were recovered from fecal sites. Only 3 patient isolates were from nonfecal sample sites (axilla). One of those patients was positive across all 3 body sites on the same day (A046). The other 2 patients (A051 and B009) both only had 1 patient isolate recovered, and it was from the axilla. Both of these isolates clustered with other occupant-stays (clusters A and F), potentially indicating pathogen movement without colonization.

### Assessing the Role of Environmental Surfaces in Pathogen Movement

While patient acquisition remained the central focus, we sought to understand all steps in the transmission pathway. Our analysis revealed an overwhelming pattern of patient isolates clustering with environmental surfaces and/or HCP hands as opposed to other patient isolates: 6 clusters (85.7%) included environmental surfaces, and 7 clusters (100%) included either environmental surfaces or HCP hands (eFigure 8 in Supplement 1). We captured the mechanism of movement of *C difficile* from 1 room to another in 5 separate instances (clusters A-C and E). We explored the sensitivity of our results to our SNV threshold in Supplement 1, eFigure 9.

We found that *C difficile* shed from patients into their own room frequently appeared on HCP hands leaving other patient rooms during concurrent stays and on other patient room surfaces during nonoverlapping stays. We did not find ample evidence of *C difficile* remaining in a room after a terminal cleaning. Only 2 of 22 clustered occupant stays (9.1%) were linked on the basis of sequential occupation of the same room.

We overwhelmingly found that isolates from the same occupant stay clustered together. However, there were 6 instances where isolates from the same occupant stay did not cluster. Two of these patients had isolates from multiple sequence types during the same occupant stay (Figure 1B): patient A250 had isolates from 2 sequence types that did not cluster with other isolates in the study, potentially indicating transmission from a patient on the ward before the start of our study; patient A046 had all but 1 isolate cluster, and the nonclustering isolate was from a HCP hand genetically identical to isolates from another cluster, indicating direct transfer between rooms. Four other occupant stays (A010, A034, B012, and B058) had isolates that varied by 7 to 53 SNVs but also did not cluster with other isolates in the study. All but 1 cluster had isolates from the same HCF. Cluster B had isolates from both HCFs: a single HCP hand isolate from hospital B clustered with isolates from hospital A.

## Discussion

In this cohort study, we quantified movement and characterized the role of environmental surfaces and HCP hands in *C difficile* transmission. Overall, we found that 7.7% of admitted patients had *C difficile* genetically linked to another occupant stay on the same ward. While this includes occupant stays with only environmental or HCP hand isolates, it reveals considerably more pathogen movement than previously recognized. This may be due in part to the extensive environmental sampling compared with other studies and/or the inclusion of nontoxigenic isolates. All of the transmission clusters we identified involved either environmental surfaces or HCP hands, leading to the identification of 3.6 times as many clusters than if we had relied solely on patient sampling, as most studies do. During the study period, only 2 patients, both in hospital B, were identified as having CDI, thus, the vast majority of isolates recovered in this study were under the radar of traditional surveillance efforts.

In contrast to many studies examining *C difficile* transmission, most isolates in this study were nontoxigenic. While nontoxigenic *C difficile* is not clinically significant, its movement in HCFs may indicate lapses in infection prevention practices and its relative abundance provides insight into transmission occurring cryptically within an HCF and serving as a model for transmission of undetected toxigenic *C difficile* as it has similar biological features (eg, durable spores).^40,41,42,43^ While toxigenic *C difficile* may be more effectively disseminated in the environment and HCP hands due to diarrheal illness, transmission of nontoxigenic *C difficile* may be more effective as contact precautions are less likely to be imposed. Therefore, transmission of nontoxigenic *C difficile,* which represents the majority of transmission in our study, may be more similar to asymptomatic toxigenic *C difficile* than to CDI transmission.

Even as our results contradict recent studies suggesting that transmission of *C difficile* may be a smaller risk than conversion from asymptomatic carriage to CDI, we believe our results underestimate the true burden of transmission within HCFs, as studies that do not track patients after transfer or discharge miss acquisitions that do not appear in stool until after hospital discharge. Since the average time from exposure to culture positivity is longer than the average length of stay for patients in the ICU, it can be challenging to detect acquisitions before patient discharge. Prior research supports the importance of inpatient hospitalization on carriage, as the strongest risk factor for *C difficile* on admission is prior hospitalization more than antibiotic use.^44^ Although our study does not add clarity to this relationship, it creates an opportunity for longer duration studies that track patients after discharge or transfer.

Our study adds important data to help explain previous findings that HCP hands and environmental surfaces are critical to transmission of HAIs.^7,11,12,45^ We identified 7 transmission clusters involving 22 occupant stays including 5 instances of contaminated HCP hands moving *C difficile* between rooms. Unlike Redmond et al,^46^ we found that in two-thirds of acquisitions, patient isolates clustered with environmental and HCP hand isolates from other occupant stays, emphasizing the contributions of these transmission pathways.

We also address the prevailing hypothesis that a patient’s room reflects the epidemiologic history of the previous occupants.^47,48^ We found 9.1% of occupant stays had isolates linked to the previous occupant stay. More frequently, we found that *C difficile* isolated from a room surface reflected *C difficile* recovered from the current occupant of the room and to a lesser extent the isolates concurrently recovered from other surfaces on the ward. Notably, pathogens from prior stays were infrequently detected in their original rooms, suggesting that *C difficile* was deposited onto surfaces and later redistributed around the HCF. As with other studies,^7,12^ we did not find evidence that a single reservoir is seeding infections; rather, transmission clusters involved a multitude of environmental surfaces. Furthermore, the extensive sampling conducted in this study coupled with the intermittent identification of *C difficile* on surfaces highlights how difficult an infectious source is to find.

### Limitations

Several limitations may affect our inferences. First, we included patients with nontoxigenic *C difficile*, most of the isolates in our study. While these primarily represent microbiota that are likely frequently exchanged between patients under standard precautions, we believe they can provide insight into how transmission of pathogens occurs that is undetected by routine surveillance measures. Another limitation is that we use a rule-based criteria to distinguish between a new acquisition and an importation, especially since daily patient body site samples were not always conducted. Patients with *C difficile* before hospitalization may initially have levels of colonization too low to produce a positive culture result but may have positive culture results once exposed to antimicrobial agents or other therapy that can promote proliferation of *C difficile*. This may be an alternative explanation for isolates that appeared during the study period but were not associated with transmission.

Next, our sampling of HCP hands was performed before glove removal or hand hygiene, potentially making it less relevant to real-world transmission. However, since most patients were not on contact precautions, for HCPs not wearing gloves, hand hygiene would most likely consist of hand sanitizing gel, which does not destroy *C difficile* spores. Additionally, we sampled only HCP hands, rather than clothing; contamination of clothing has been documented with other pathogens and may be a potentially important mechanism for *C difficile* spread.^3,6,7^

## Conclusions

In conclusion, our study is among the first to provide direct genomic evidence for the role of environmental surfaces and HCP hands in *C difficile* transmission within an HCF. We demonstrated substantially more *C difficile* movement than had been previously appreciated. Although we highlight the role of environmental surfaces in transmission, we did not find a single reservoir responsible for seeding infections across the wards. Our observations provide novel insights into transmission dynamics that can inform infection control practices by highlighting the critical importance of hand hygiene, even when CDI is not suspected. Furthermore, our results suggest that studies that rely solely on patient sampling may underestimate transmission because patients on average have very short lengths of stay. This underscores the need for future studies to track patients as they move through the health care network to identify potential patient linkages for those who may have acquired *C difficile* but are discharged before they begin shedding it into the environment.

## Notes

Supplement 1.
eMethods.
**eFigure 1. **Flow chart of sampling and isolate recovery
eResults.
**eFigure 2.** Heatmap of the number of samples and number of *C difficile* isolates by sampling location **eFigure 3.** *C difficile* prevalence and sequence type diversity within and between patients over a 127-day period in 2018 **eFigure 4.** Phylogenetic tree of the *C difficile* isolates from 2 HCFs **eFigure 5. **Plot of within-host variation between the first isolate recovered from a given occupant stay and all future isolates collected **eFigure 6.** Plot of the mean genomic distances (in SNVs) between granular sampling locations within an occupant stay **eFigure 7.** Plot of number of days from first patient isolate to first HCP hand or room environment isolate for a given occupant stay **eFigure 8. **Epidemiologic timing of samples and isolates in the transmission clusters **eFigure 9. **Plot of SNV threshold-based clusters coupled with timing of sample collection for the isolates in each cluster for relaxed threshold

Supplement 2.
Data Sharing Statement
