# Supplementary material for: Environmental and Health Care Personnel Sampling and Unobserved Clostridium difficile Transmission in ICU
Source: JAMA Netw Open. 2025 Apr 4;8(4):e252787. doi: 10.1001/jamanetworkopen.2025.2787 (PMC11971673; doi:10.1001/jamanetworkopen.2025.2787)
Supplement: Supplement 1. — eMethods. eFigure 1. Flow chart of sampling and isolate recovery eResults. eFigure 2. Heatmap of the number of samples and number of C difficile isolates by sampling location eFigure 3. C difficile prevalence and sequence type diversity within and between patients over a 127-day period in 2018 eFigure 4. Phylogenetic tree of the C difficile isolates from 2 HCFs eFigure 5. Plot of within-host variation between the first isolate recovered from a given occupant stay and all future isolates collected eFigure 6. Plot of the mean genomic distances (in SNVs) between granular sampling locations within an occupant stay eFigure 7. Plot of number of days from first patient isolate to first HCP hand or room environment isolate for a given occupant stay eFigure 8. Epidemiologic timing of samples and isolates in the transmission clusters eFigure 9. Plot of SNV threshold-based clusters coupled with timing of sample collection for the isolates in each cluster for relaxed threshold [file jamanetwopen-e252787-s001.pdf]

Supplemental Online Content

Keegan LT, Tanner W, Orleans B, et al. Environmental and healthcare personnel sampling and unobserved *C. difficile* transmission in ICU. *JAMA Netw Open*. 2025;8(4):e252787. doi:10.1001/jamanetworkopen.2025.2787

eMethods

eFigure 1. Flow chart of sampling and isolate recovery

eResults.

eFigure 2. Heatmap of the number of samples and number of C. difficile isolates by sampling location

eFigure 3. C. difficile prevalence and sequence type diversity within and between patients over a 127-day period in 2018

eFigure 4. Phylogenetic tree of the C. difficile isolates from 2 HCFs

eFigure 5. Plot of within-host variation between the first isolate recovered from a given occupant stay and all future isolates collected

eFigure 6. Plot of the mean genomic distances (in SNPs) between granular sampling locations within an occupant stay

eFigure 7. Plot of number of days from first patient isolate to first HCP hand or room environment isolate for a given occupant stay

eFigure 8. Epidemiologic timing of samples and isolates in the transmission clusters

eFigure 9. Plot of SNP threshold-based clusters coupled with timing of sample collection for the isolates in each cluster for relaxed threshold

This supplemental material has been provided by the authors to give readers additional information about their work.

© 2025 Keegan LT et al. *JAMA Network Open*.

eMethods

Sampling: Patients were sampled using sterile flocked Eswabs (ThermoFisher Scientific ESwab™ Waltham, MA) moistened with transport media. Patients were able to consent to any or all body sites and were able to withdraw consent throughout the study. Premoistened sponge-wipes (3M™ Sponge-Stick, St. Paul, MN) were used to collect samples from hospital room environmental surfaces.

For patients not on contact precautions, these samples were a composite of surfaces in three zones: Zone 1 included the near-patient surfaces such as the bed rails and beside table; Zone 2 included the HCP touch areas such as the computer, IV pole, and supply cabinet; and Zone 3 included the toileting areas which included toilet grab bars, flush handle, rinse spout or commode handles if commode present. For patients who were on contact precautions, samples were collected from individual surfaces rather than as a composite. The five surfaces sampled from the environment of patients on contact precautions were: bed rails, over-bed table, door handle, door grab areas, HCP touch areas (same surfaces as Zone 2), and the toileting area (same surfaces as Zone 3). Hands or gloves (if worn) of HCPs who cared for the patient were also sampled upon exit from the patient's room and before HCPs completed hand hygiene or glove removal. At least one HCP hand sample was collected from each occupant room, each day as HCP was leaving the patient room. Shared surfaces were sampled daily (Figure S1).

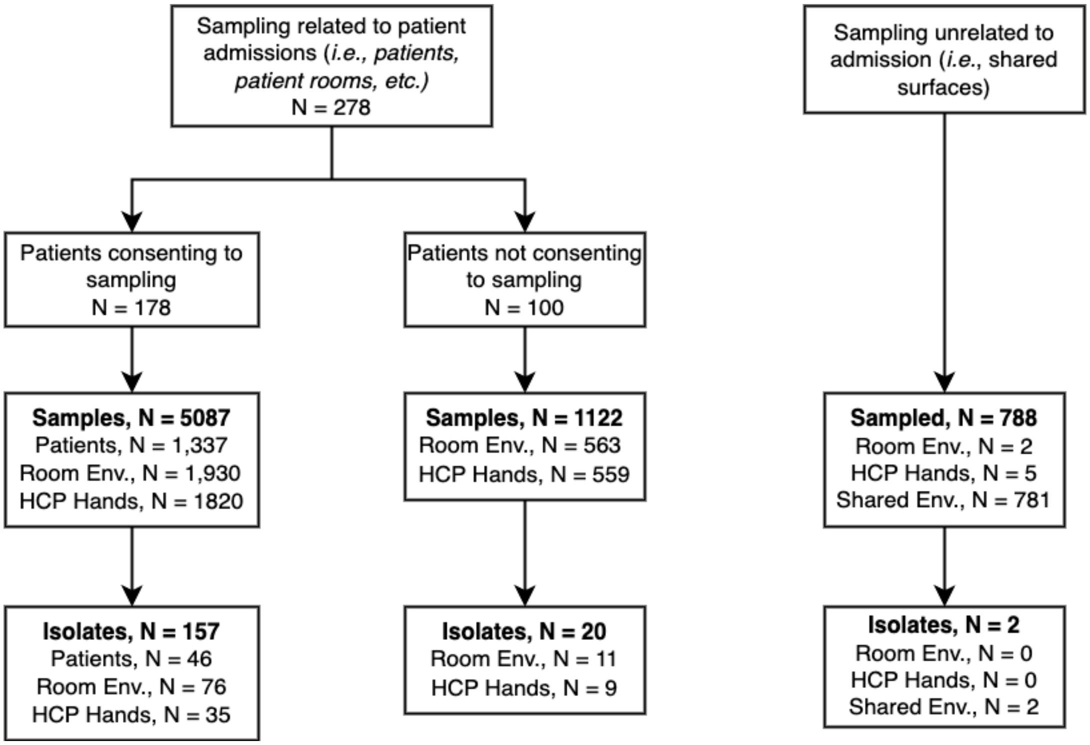

© 2025 Keegan LT et al. *JAMA Network Open*.

eFigure 1. Flow chart of sampling and isolate recovery. Sampling followed two streams: sampling related to patient admissions, such as patient body sites, patient room environmental surfaces, and HCP hands leaving occupied rooms or unrelated to patient surfaces, this included shared environmental surfaces, and empty, unoccupied patient room surfaces. Sampling related to patient admissions was split again based on patients consenting to sampling. Patients who consented to sampling were sampled daily in three body sites if they were in their room at the time of sampling at the same time, patient rooms and HCP hands were sampled. If a patient was not in their room during sampling, their room was still sampled. For patients who did not consent to sampling, their room and HCP hands were sampled. While sampling unrelated to patient admissions was primarily on shared surfaces, in some cases, empty, unoccupied rooms were sampled.

Occupant Stay ID assignment: Unique occupant stay IDs were assigned sequentially from admission and we used the numbering to denote whether patient sampling was conducted. Unique occupant stay IDs numbered from 001 – 199 indicate that the patient consented to patient sampling and IDs numbered from 200 –399 indicate that the patient did not consent to patient sampling and thus only environmental and HCP hand samples were collected. We also assigned a unique occupant stay ID to vacant rooms, these IDs range from 900 – 1000 and included any samples collected while the room was empty, from when the previous patient was discharged and until the next patient was admitted.

Microbiologic Testing: Organisms were eluted from sponge-wipes in phosphate-buffered saline with 0.22% Tween®80 using a homogenizer (Stomacher®400 Circulator; Seward Laboratory systems, Inc.).[20] Transport media with ESwabs® were vortexed. Swab transport media and sponge eluates were plated to *C. difficile* CCFA-HT agar or CCMB-TAL broth. Positive CCMB-TAL tests were subcultured to CCFA-HT.[20] Matrix-assisted laser desorption-ionization time of flight mass spectrometry (MALDI - TOF) is a rapid method for identifying microorganisms based on the molecular weight of proteins specific to each organism. A portion of an isolated colony was directly spotted onto a target and covered with α-Cyano-4-hydroxycinnamic acid matrix. The prepared target was placed into the mass spectrophotometer and was hit with a finely directed laser beam, which vaporizes and ionizes the proteins in the sample. The ionized proteins were accelerated in a vacuum flight tube, which separates them based on size. The time it takes for particles to reach a detector at the end of the tube was measured and used to generate a spectrum for each tested organism. The spectrum of the unknown organism is compared to a library of spectra from known organisms and a probability of a given identification is assigned. For *C. difficile*, identification was reported at the species level when the probability score value was $\geq$ 2.0.

© 2025 Keegan LT et al. *JAMA Network Open*.

Period Prevalence Calculation: We calculated the overall period prevalence as the number of occupant stays with *C. difficile* isolated from that location (*i.e.*, body site, environment, HCP hands) compared to the total number of occupant stays with sampling from that location.

Laboratory cross-contamination: Laboratory cross-contamination was found to have occurred in samples from Hospital A during a 3-week period when some sponge samples from environmental and HCP hands were contaminated with *Pseudomonas proteolytica* and other non-fermenting Gram-negative rods. Patient samples were free from cross-contamination. A total of 4 *C. difficile* isolates were potentially affected, and these samples are included in our analysis.

Bioinformatics: We prepared the genomes for assembly by trimming adapters and phiX with bbduk[23] and poor-quality sequences using seq-qc. [24] De novo assemblies were constructed with SPAdes[25] and annotated with prokka.26 As an assembly validation check, the original sequencing reads were mapped to each assembly with bowtie2,27 and all assemblies had similarly low rates of mismatches between mapped reads and the assembled scaffolds.

Phylogenetic Tree Construction: Genomes were assigned to a clade according to their similarities to each reference as calculated by fastANI.30 For each clade, a recombination-corrected, maximum likelihood phylogeny was inferred from the whole-genome core alignment using Gubbins and RAxML.31,32 Support for each branch was calculated using bootstrapping. The phylogenetic trees were visualized using the ggtree package in R .33–35,37 Genomic distances between each genome and its clade reference were calculated from the recombination-corrected alignment with snp-dists.36

Genomic Distance Evaluation: To quantify the within- and between-facility as well as the within- and between-occupant stay genomic distances, we calculated the mean, standard deviation, and confidence intervals of pairwise genomic distances for all isolates collected in each hospital.

eResults

Cohort description: As part of a longitudinal, observational study conducted across two ICUs, we collected daily samples from three patient body sites (axilla, groin, and perianal), surfaces in three patient room environmental areas (near bed, far bed, and toilet area), HCP hands prior to hand hygiene or glove removal, and shared environmental surfaces outside patient rooms (Figure S2). We collected a total of 7,000 samples, of which 19.1%

© 2025 Keegan LT et al. *JAMA Network Open*.

were from patient body sites, 35.6% from patient room surfaces, 11.1% from shared environmental surfaces, and 34.1% from HCP hands.

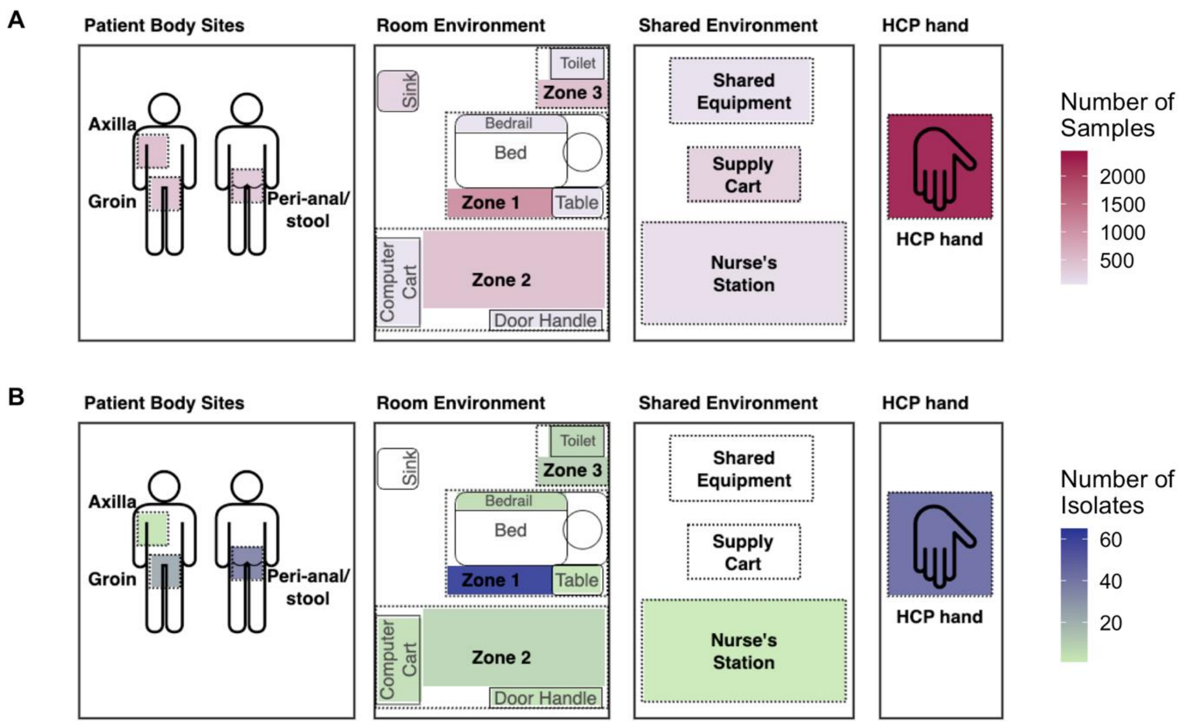

eFigure 2. Heatmap of the number of samples and number of *C. difficile* isolates by sampling location. Each box represents a different sampling environment (patient body sites, room environment surfaces, shared environmental surfaces, and HCP hands. **A)** shows the number of samples collected by location and **B)** shows the number of *C. difficile* isolates recovered from each sampling environment. White indicates that no *C. difficile* isolates were recovered.

Across both ICUs, 178 unique admissions consented to patient body site sampling. While a similar number of patients consented to sampling in both hospitals , these represent a different percent of the overall patients, 61.5% and 72.4% of all patients in hospital A and hospital B, respectively. Likewise, 66.6% and 84.6% of patients with at least one *C. difficile* isolate consented to sampling in Hospital A and B, respectively.

The median number of samples collected per occupant stay was 14 (interquartile range [IQR]: 7 – 23). Among the 177 unique occupant stays during which body sites were sampled, *C. difficile* was identified from patient body sites in 12 (6.8%) samples, the environment in 14 (7.9%) samples, and HCP hands in 15 (8.5%) samples. The recovery of

© 2025 Keegan LT et al. *JAMA Network Open*.

C. difficile from a patient body site was strongly associated with its recovery from room surfaces (odds ratio [OR], 12.07 95% confidence interval [CI] 2.52–55.75, p < 0.005) and from HCP hands (OR, 15.46 95% CI: 2.57–89.47, p<0.005).

We found the median length of stay was significantly longer for occupant stays with at least one *C. difficile* isolate (4 days [IQR: 3.0 – 15.50 days]) compared to occupant stays without *C. difficile* (2 days [IQR: 1–3 days]) (Wilcoxon rank sum p <0.001).

An examination of period prevalence revealed the combined period prevalence of *C. difficile* (toxigenic and non-toxigenic) among patient body sites was 5.23% at hospital A and 9.52% at hospital B. The combined period prevalence of *C. difficile* (toxigenic and non-toxigenic) for environmental surfaces was 6.41% at hospital A and 9.20% at hospital B. Similarly, the period prevalence of toxigenic *C. difficile* alone among patient body sites was 0% at hospital A and 4.76% at hospital B. The period prevalence of toxigenic *C. difficile* alone among environmental surfaces was 3.74% at hospital A and 2.30% at hospital B.

Quantification of ST diversity: We assessed the diversity of strains in our study at the sequence type (ST) level to examine spatial or temporal trends in ST diversity as well as to enable comparison with previous studies (Figure S3). We found ST 15 was the most common (34.5%), followed by ST 3 (29.9%), ST 2 (10.7%), and ST 39 (9.6%). We constructed a phylogenetic tree of the *C. difficile* isolates collected in this study and found isolates from clades 1, 2, and 4 (Figure S4). All toxigenic isolates were positive for both *tdcA* and *tdcB* genes.

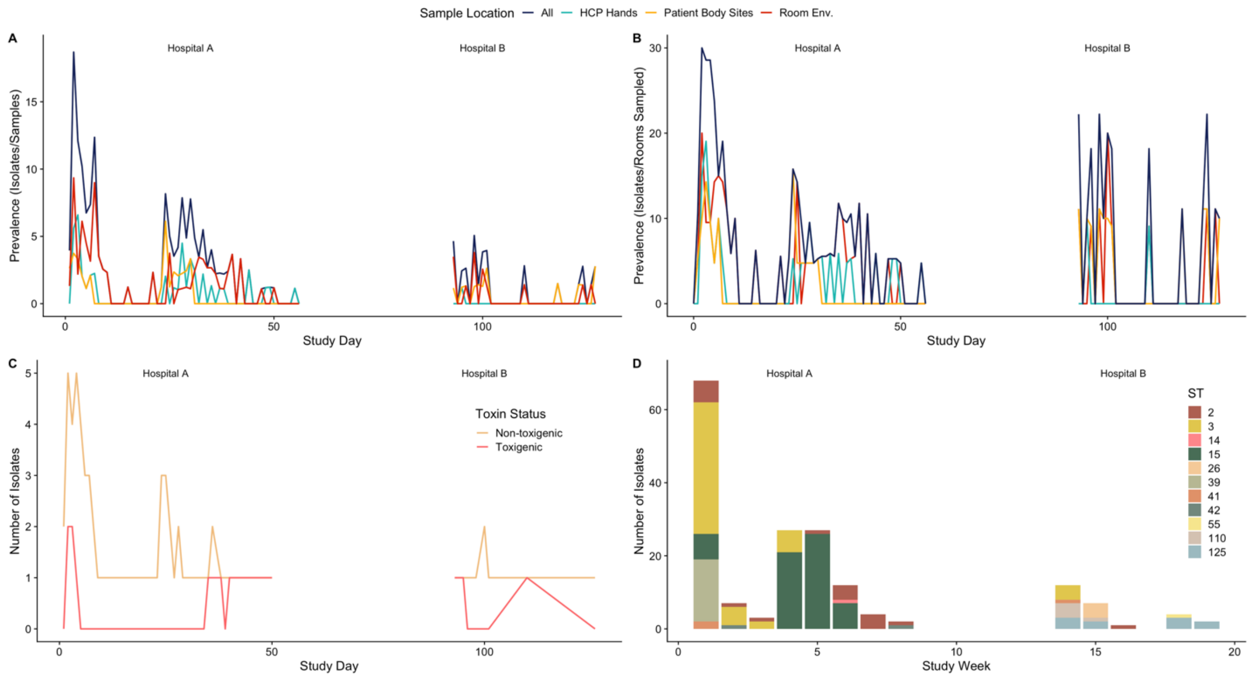

© 2025 Keegan LT et al. *JAMA Network Open*.

eFigure 3. *C. difficile* prevalence and sequence type diversity within and between patients over a 127-day period in 2018. A) shows the percent of *C. difficile* isolates among samples for all sampling locations for each day in both hospitals. **B)** shows the percent of *C. difficile* isolates among rooms for all sampling locations for each day in both hospitals. **C)** shows the daily number of unique occupant-stays with at least one sample positive for *C. difficile*. **D)** shows the weekly distribution of sequence type for isolates across each hospital.

Longitudinal comparison of C. difficile diversity across scales

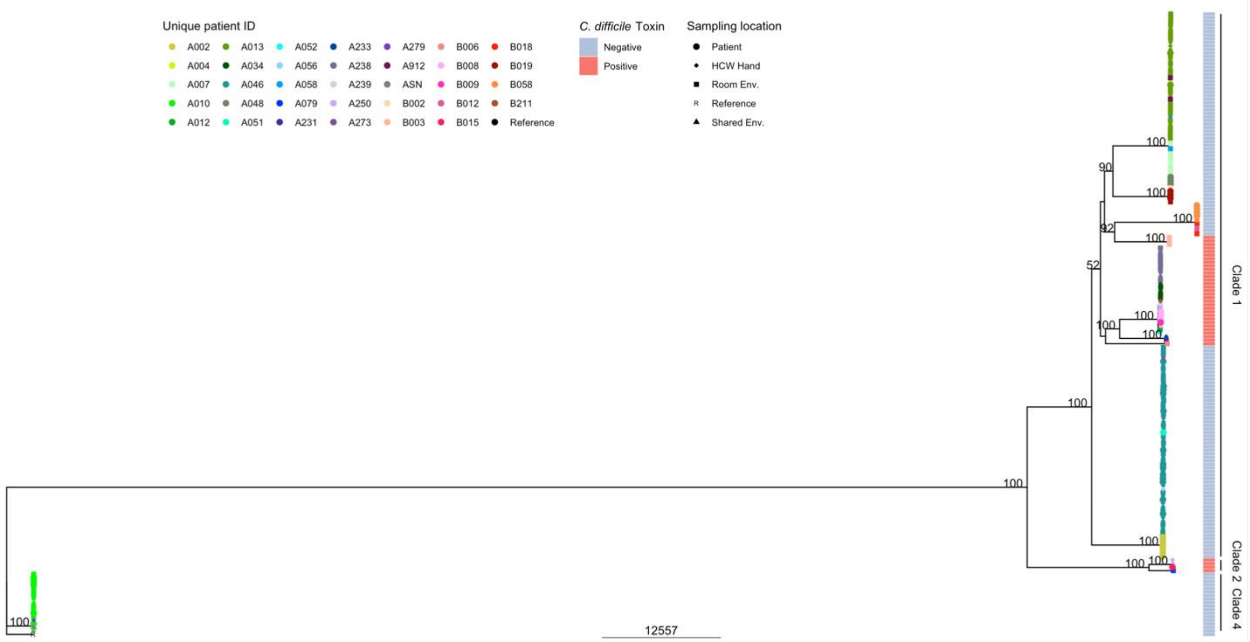

eFigure 4. Phylogenetic tree of the C. difficile isolates from 2 HCFs. Each tip represents the genomic sequence from samples collected from any surface, during an individual occupant stay. Tips colors represent the occupant stay. The location within the room where the isolate was collected is depicted by the shape. Bootstrap values with >50% support are shown for major branches. Whether or not an isolate was toxigenic is mapped onto the tree as a heat map.

Through sequential sampling within an individual occupant stay, we compared the first isolate, regardless of location, to all other isolates from that occupant stay and as expected we found no pattern indicative of evolution over an occupant's stay (Figure S5). Within an

© 2025 Keegan LT et al. *JAMA Network Open*.

occupant stay, we found the genomic distance was generally low and consistent with long-term carriage, as expected given the slow molecular clock of *C. difficile*.[26]

Similar to our finding that isolates from patient body sites were more closely related to isolates from the patients' own room than to isolates from other patients or different rooms, we found isolates from environmental surfaces and HCP hands were most genetically similar to isolates from their same occupant stay (mean 0.48 SNPs [IQR:0–0] and 43.8 SNPs [IQR:0–0], respectively) compared to isolates from other occupant stays (mean 2054 SNPs [IQR: 964–1285] and 1828 SNPs [IQR: 964–1898], respectively). Though not significant, we found that isolates collected from HCP hands show the greatest within-occupant stay genetic diversity between contact and standard precautions occupant stays (mean 0 SNPs [IQR:0–0] and 103 SNPs [IQR:0–0], respectively).

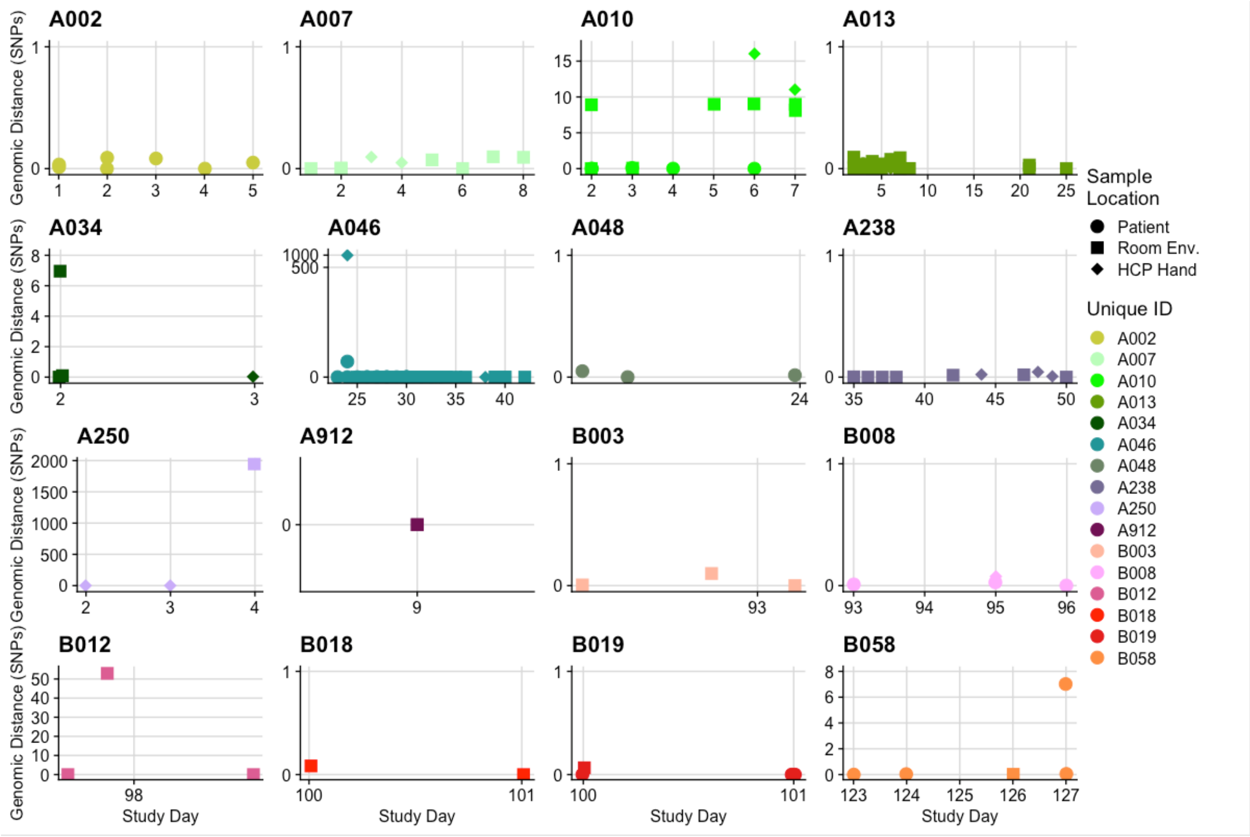

eFigure 5. Plot of within-host variation between the first isolate recovered from a given occupant stay and all future isolates collected. For all patients with at least two *C. difficile* isolates recovered, we show the number of SNPs between the first isolate recovered and all subsequent isolates recovered.

© 2025 Keegan LT et al. *JAMA Network Open*.

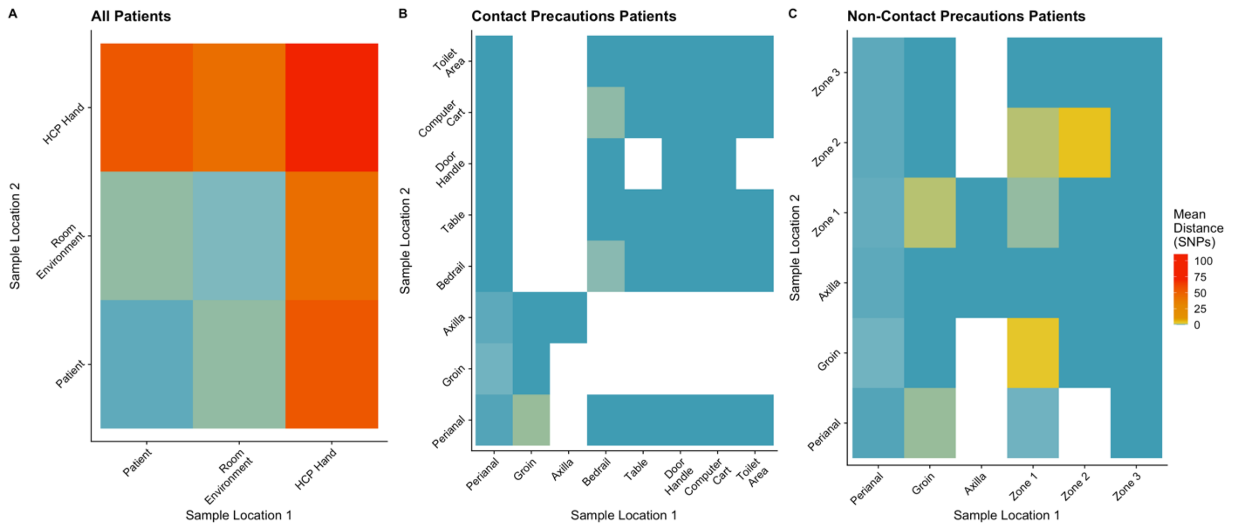

eFigure 6. Plot of the mean genomic distances (in SNPs) between granular sampling locations within an occupant stay. A) shows the average distance between all pairs of isolates within an occupant stay, by sample location. **B)** shows the average distance between all pairs of patient, room environment, and HCP hand isolates within an occupant stay for patients on contact precautions, including isolates from patient body sites. **C)** shows the average distance between all pairs of patient, room environment, and HCP hand isolates within an occupant stay for patients not on contact precautions.

Quantification of C. difficile importation and acquisition: We estimated the importation frequency by defining an importation as detection of *C. difficile* colonization on admission or the next calendar day. We found 2 patients imported toxigenic and 5 imported non-toxigenic *C. difficile* (Figure 3). One patient in our study met the criteria for an acquisition, as we did not recover *C. difficile* on admission or the following day and found they were colonized on the third day of ICU stay or later. Three patients did not meet the criteria because they were not tested on admission or the next day, and 12 patients were tested but C. difficile was never recovered. For the 4 patients who had *C. difficile* recovered from environmental or HCP hand samples and patient samples, the first room environmental sample from which C. difficile was recovered was 0.5 days after the occupying patient's first isolate, while the first recovery from HCP hand samples was 1.5 days after (Figure S7).

© 2025 Keegan LT et al. *JAMA Network Open*.

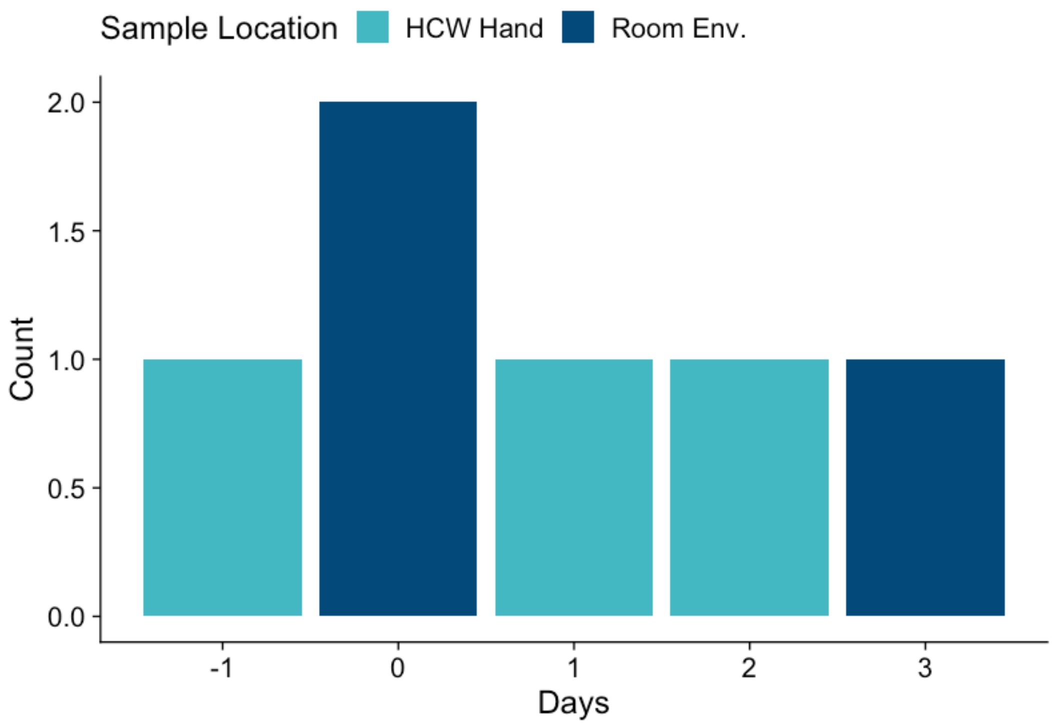

eFigure 7. Plot of number of days from first patient isolate to first HCP hand (teal) or room environment isolate (dark blue) for a given occupant stay.

Assessing the role of environmental surfaces in pathogen movement: Our findings revealed patterns in the timing of potential sources only a single isolate is recovered over the entire occupant stay. Assuming these occupant stays with transient *C. difficile* (defined as occupant stays with only one day of *C. difficile* isolates) were likely spread from occupant stays where we persistently recovered *C. difficile* (defined as occupant stays with at least 2 days of *C. difficile* isolates), we found a median of 1.5 days to the temporally closest potential transmission source. This result is highly variable by sample location: the median time to the most recent source for room environment isolates was 7 days and only 0.5 days to the most recent source for HCP hand isolates.

Genomic analysis of pathogen movement in the ICU: In all but one instance (Figure 4 Cluster B), clusters were transitive, where all isolates were within the threshold of all other isolates in the cluster.

© 2025 Keegan LT et al. *JAMA Network Open*.

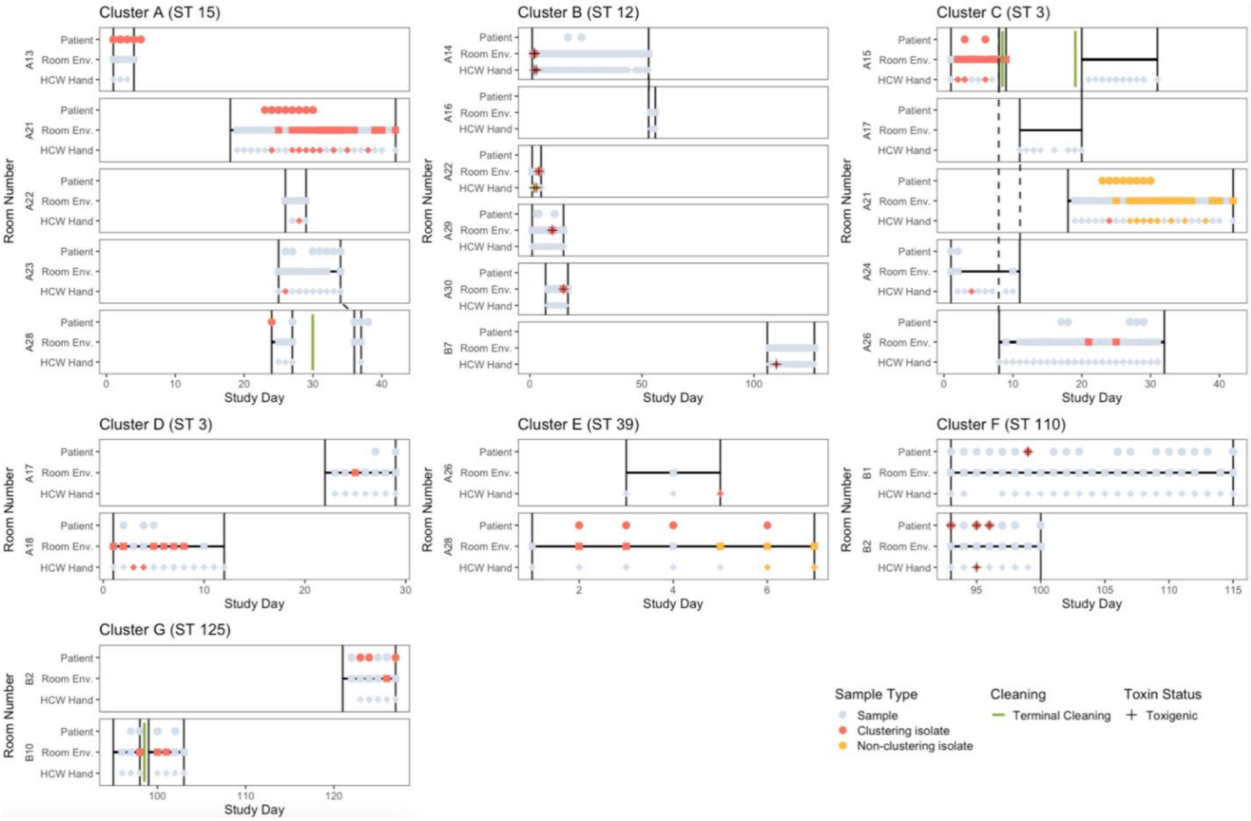

eFigure 8. Epidemiologic timing of samples and isolates in the transmission clusters. Plot of isolates that cluster with other isolates in our study and detail on the clusters. Each sub plot represents a transmission cluster (A–G) where each facet plot represents a room in the cluster and the labels are colored according to cluster ST. Inside each facet plot, each point represents a collected sample and point color indicates isolate clustering. Black horizontal lines connected to vertical bars indicate room transfers for patients in the cluster and terminal room cleanings are only in indicated where multiple unique occupant stays were sequentially in the same room, otherwise terminal cleanings are implied. All isolates in Clusters B and F are toxigenic.

© 2025 Keegan LT et al. *JAMA Network Open*.

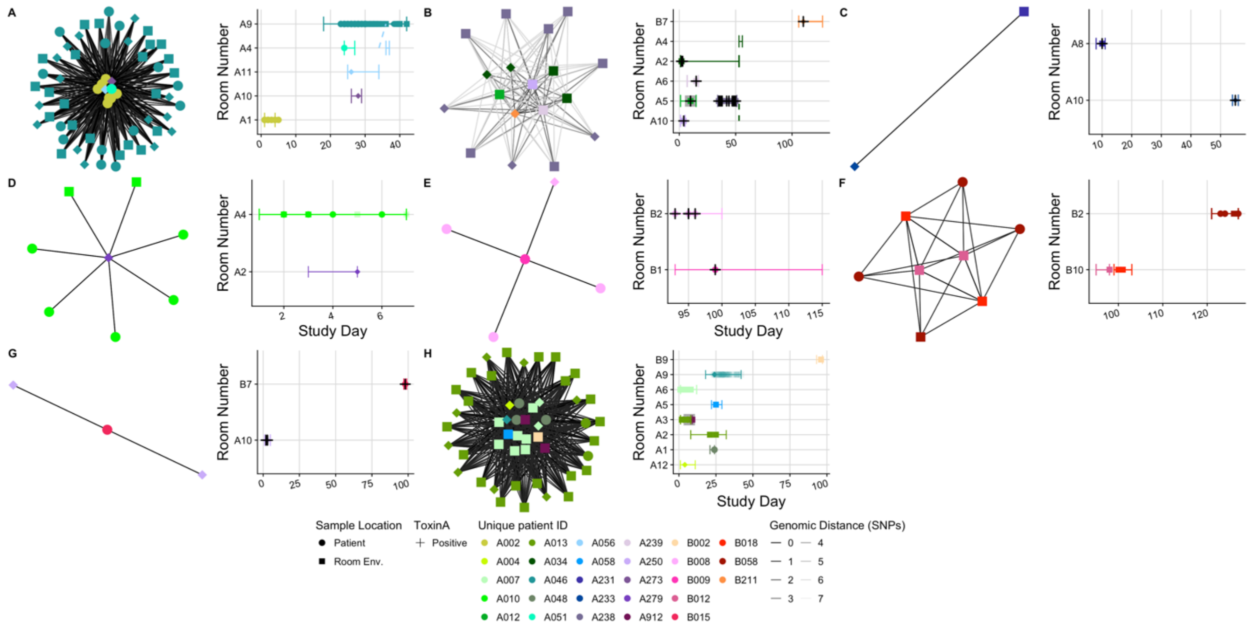

eFigure 9. Plot of SNP threshold-based clusters coupled with timing of sample collection for the isolates in each cluster for relaxed threshold. Network plot of each cluster with each isolate represented as a node and an edge for each connection between two distinct occupant stays. The color of each node is given by the occupant stay ID and the shape is given by the sampling location, with circles representing patients, squares representing room environments, diamonds representing HCP hands, and triangles representing shared environmental surfaces. The color of each edge is given by the distance (in SNPs) between any pair of isolates with ranging from black (0 SNPs) to light grey (7 SNPs). Each cluster is accompanied by a descriptive figure of the collection dates and room locations of the isolates in the cluster as well as the admission, discharge, and time on the ward (vertical lines connected by horizontal lines). Points that are full opacity indicate the isolates from an occupant stay that are included in the cluster while points at partial opacity (*e.g.*, A046 in cluster C) indicate other isolates collected from the same occupant stay that do not cluster. Points that occur after the discharge date (*e.g.*, A002 in cluster A) indicate follow up sampling after a patient was transferred to another unit in the same hospital.

Clustering threshold sensitivity analysis: While molecular clock data[49], similar studies,[3,6,7] and our data support a clustering threshold of ≤2 SNPs, in a sensitivity analysis, we explored loosening our SNP-threshold to ≤7 SNPs. This less restrictive threshold revealed that 4 of the 7 clusters do not change; 2 clusters clustered together; and 2 new clusters formed, both with a long time-lag between sample collection (Figure S9 G, H). We explored loosening our SNP-threshold from ≤2 SNPs to ≤7 SNPs to move from capturing 98.1% of all pairwise distances between isolates from the same patient to capturing 100% of all within-patient distances. Reconstructing transmission clusters for all pairs of isolates with ≤7 SNPs revealed that 4 of the 7 clusters formed with a threshold of ≤2 SNPs do not

change. One of the original clusters loses directionality (*i.e.*, all isolates cluster with all other isolates) (Figure 4B, Figure S9B) and 2 of the original clusters merged into 1 (Figure 4C, D, Figure S9 C). Two new clusters are formed when a threshold of ≤7 SNPs is chosen, however both have a long time-lag between sample collection dates (Figure S9 B, G). Other studies that have relaxed their ≤2 SNP threshold used at ≤5 SNP threshold. We also explored a ≤5 SNP threshold and found it produced the same clusters as the ≤7 SNP threshold.

© 2025 Keegan LT et al. *JAMA Network Open*.
