# Supplementary material for: Environmental and Health Care Personnel Sampling and Unobserved Clostridium difficile Transmission in ICU
Source: JAMA Netw Open. 2025 Apr 4;8(4):e252787. doi: 10.1001/jamanetworkopen.2025.2787 (PMC11971673; doi:10.1001/jamanetworkopen.2025.2787)
Supplement: Supplement 2. — Data Sharing Statement [file jamanetwopen-e252787-s002.pdf]

Data Sharing Statement

Keegan. Environmental and Healthcare Personnel Sampling and Unobserved C. Difficile Transmission in ICU. *JAMA Netw Open*. Published April 01, 2025.
doi:10.1001/jamanetworkopen.2025.2787

Data
Data available: Yes
Data types: Deidentified participant data, Data (not involving human participants)
How to access data: Whole genome sequence data are available here:
https://www.ncbi.nlm.nih.gov/bioproject/968176
When available: With publication

Supporting Documents
Document types: Statistical/analytic code
How to access documents: code is available here:
https://github.com/Keeganlt/CdifficileTransmissionClustering
When available: With publication

Additional Information
Who can access the data: Data is publicly available.
Types of analyses: Data is publicly available.
Mechanisms of data availability: Data is publicly available.
Any additional restrictions: Data is publicly available.
